# Nutrition state of science and dementia prevention: recommendations of the Nutrition for Dementia Prevention Working Group

**DOI:** 10.1016/s2666-7568(22)00120-9

**Published:** 2022-07-04

**Authors:** Hussein N Yassine, Cécilia Samieri, Gill Livingston, Kimberly Glass, Maude Wagner, Christy Tangney, Brenda L Plassman, M Arfan Ikram, Robin M Voigt, Yian Gu, Sid O’Bryant, Anne Marie Minihane, Suzanne Craft, Howard A Fink, Suzanne Judd, Sandrine Andrieu, Gene L Bowman, Edo Richard, Benedict Albensi, Emily Meyers, Serly Khosravian, Michele Solis, Maria Carrillo, Heather Snyder, Francine Grodstein, Nikolaos Scarmeas, Lon S Schneider

**Affiliations:** Department of Medicine (H N Yassine MD) and Department of Neurology (H N Yassine, Prof L S Schneider MD MS), Department of Psychiatry and Neuroscience (Prof L S Schneider), and Department of Gerontology (Prof L S Schneider), Keck School of Medicine and Department of Medicine (S Khosravian BA), University of Southern California, Los Angeles, CA, USA; Bordeaux population health U1219, National Institute of Health and Medical Research (INSERM)—University of Bordeaux, Bordeaux, France (C Samieri PhD); Division of Psychiatry, University College London, London, UK (G Livingston MD); Camden and Islington NHS Foundation Trust, London, UK (G Livingston); Channing Division of Network Medicine, Brigham and Women’s Hospital, Boston MA, USA (K Glass PhD); Department of Medicine, Harvard Medical School (K Glass) and Department of Biostatistics, Harvard Chan School of Public Health (K Glass), Harvard University, Boston MA, USA; Rush Alzheimer’s Disease Center (M Wagner PhD, F Grodstein ScD), Departments of Clinical Nutrition and Preventive Medicine (C Tangney PhD), Rush Center for Microbiome and Chronobiology Research (R M Voigt PhD), Department of Internal Medicine (R M Voigt), and Department of Anatomy and Cell Biology (R M Voigt), Rush University Medical Center (M Wagner) and Department of Neurological Sciences (M Wagner), Rush Medical College, Rush University, Chicago IL, USA; Department of Psychiatry and Behavioral Sciences, Duke University, Durham NC, USA (B L Plassman PhD); Department of Epidemiology, Erasmus MC University Medical Center, Rotterdam, Netherlands (M A Ikram MD PhD); Department of Neurology and Department of Epidemiology, Taub Institute, Sergievsky Center, Columbia University Irving Medical Center (Y Gu MD PhD), and Department of Neurology (N Scarmeas MD), Colombia University, New York, NY, USA; University of North Texas Health Science Center, University of North Texas, Fort Worth, Texas TX, USA (S O’Bryant PhD); Norwich Medical School (A M Minihane PhD) and Norwich Institute of Healthy Ageing (A M Minihane), University of East Anglia, Norwich, UK; Department of Internal Medicine-Geriatrics, Wake Forest University School of Medicine, Wake Forest University, Wake Forest, NC, USA (S Craft PhD); Geriatric Research Education and Clinical Center, Minneapolis VA Health Care System, Minneapolis, MN, USA (H A Fink MD MPH); Biostatistics School of Public Health, University of Alabama at Birmingham, Birmingham AL, USA (S Judd PhD MPH); Aging Research team, Centre for Epidemiology and Research in Population Health, INSERM (S Andrieu MD PhD) and Department of Clinical Epidemiology and Public Health, University of Toulouse Hospital, University of Toulouse III—Paul Sabatier, Toulouse, France (S Andrieu); NIA-Layton Aging and Alzheimer’s Disease Research Center, Department of Neurology, Oregon Health and Science University, Portland OR, USA (G L Bowman ND MPH); Helfgott Research Institute, National University of Natural Medicine, Portland OR, USA (G L Bowman); Department of Neurology, Donders Institute from Brain, Behavior and Cognition, Radboud University Medical Centre, Nijmegen, Netherlands (E Richard MD PhD); Department of Public and Occupational Health, Amsterdam University Medical Centre, University of Amsterdam, Amsterdam, Netherlands (E Richard); Department of Pharmaceutical Sciences, College of Pharmacy, Nova Southeastern University, Davie FL, USA (B Albensi PhD); St Boniface Hospital Research Center, Winnipeg MB, Canada (B Albensi); Department of Pharmacology and Therapeutics, University of Manitoba, Winnipeg MB, Canada (B Albensi); Alzheimer’s Association, Chicago, IL, USA (E Meyers PhD, M Solis PhD, M Carrillo PhD, H Snyder PhD); Department of Neurology, Aiginitio Hospital, Medical School, National and Kapodistrian University of Athens, Athens, Greece (N Scarmeas)

## Abstract

Observational studies suggest that nutritional factors have a potential cognitive benefit. However, systematic reviews of randomised trials of dietary and nutritional supplements have reported largely null effects on cognitive outcomes and have highlighted study inconsistencies and other limitations. In this Personal View, the Nutrition for Dementia Prevention Working Group presents what we consider to be limitations in the existing nutrition clinical trials for dementia prevention. On the basis of this evidence, we propose recommendations for incorporating dietary patterns and the use of genetic, and nutrition assessment tools, biomarkers, and novel clinical trial designs to guide future trial developments. Nutrition-based research has unique challenges that could require testing both more personalised interventions in targeted risk subgroups, identified by nutritional and other biomarkers, and large-scale and pragmatic study designs for more generalisable public health interventions across diverse populations.

## Introduction

As the number of people with dementia increases worldwide, dementia remains largely untreatable and incurable. However, the development of dementia can be delayed or even prevented, as numerous modifiable risk factors have been identified.^1^ These include hypertension, obesity, smoking, and physical inactivity—risk factors long recognised as detrimental for general health. Many of the factors are inter-related, which means that even targeting one could lead to a cascade of benefits for a person at risk for dementia.^2^

A healthy diet is fundamental to healthy living, and good nutrition can reduce incidence of diseases that are themselves risk factors for dementia, such as hypertension and type 2 diabetes.^3^ However, the data establishing associations between nutrition and cognitive health remain inconclusive.^4^ Research on diet and health conditions such as hypertension or heart disease has a long history but our understanding of how diet affects cognition is still developing, with mixed, and sometimes inconsistent, results.^1^ In 2020, *the Lancet Commission on Dementia* did not include diet in its list of modifiable risk factors associated with dementia.^1^ Diet has been considered to be a single risk-protective factor, but in fact, in contrast to most other factors (eg, smoking, or hypertension), diet is a multidimensional exposure that encompasses multiple healthy and unhealthy elements, provided by food and beverages under specific habits, and sometimes as part of a multimodal constellation of lifestyle factors.

**Figure 1:**
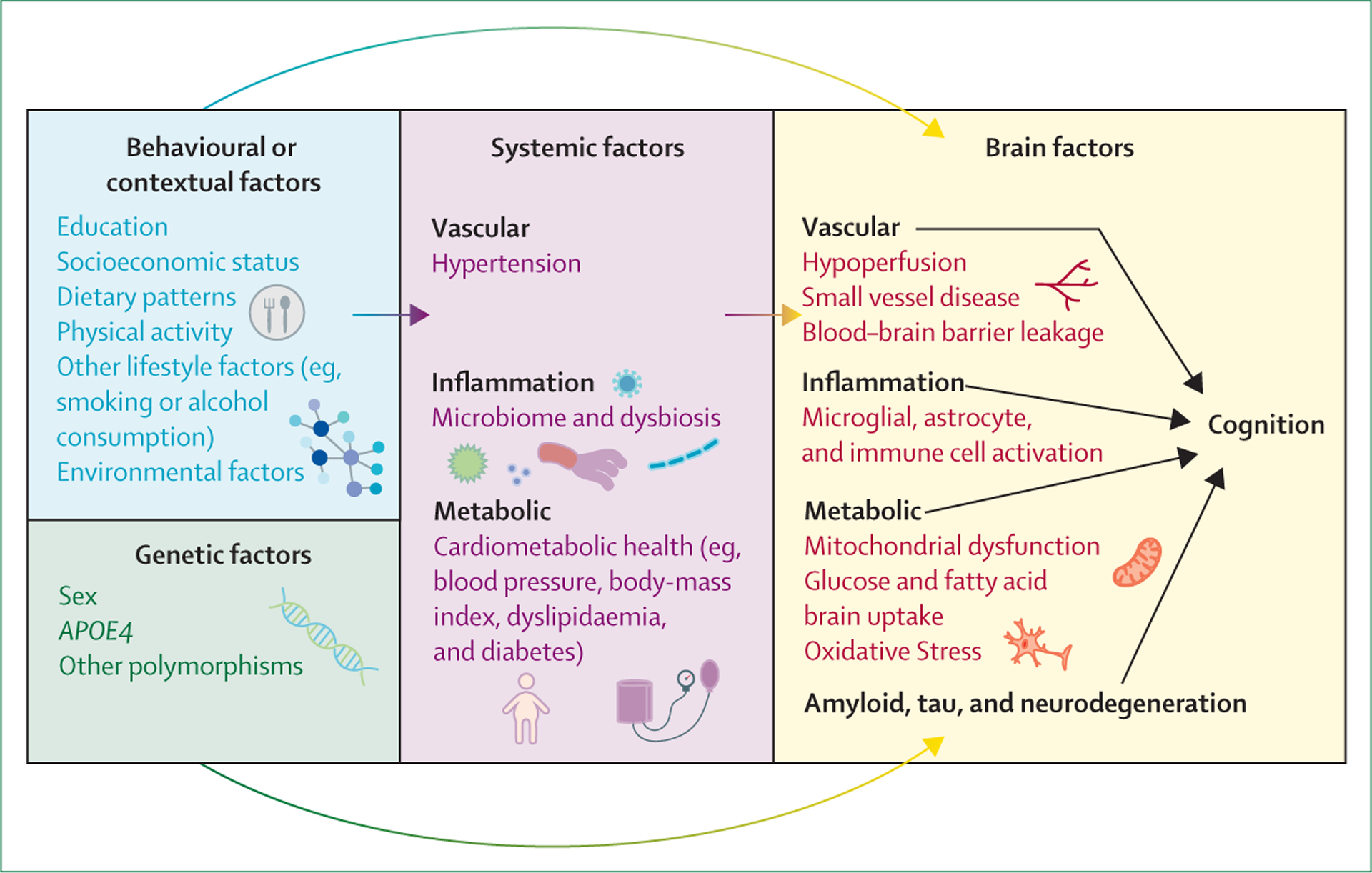
Biological pathways mediating the relationship of the diet with cognition The effect of the diet on cognition involves complex interactions that include behavioural, genetic, systemic, and brain factors. The diet can affect the brain directly or indirectly through chronic diseases (dementia risk factors). The blood-brain barrier has pleiotropic functions that include nutrient brain delivery, and a leaky blood-brain barrier in Alzheimer’s disease is associated with brain glucose hypometabolism.

The issue of whether the effects of nutrition on the brain are independent or correlates of other healthy behaviours remains open to debate. The biological pathways mediating the relationship between diet and cognition involve both direct and indirect effects on the brain (figure 1). Although important from a public health point of view, interventions of diet since 2010, along with other lifestyle changes,^5–7^ have not addressed whether the effects are independent or correlates. Previous epidemiological observations examining diet in relation to physical activity has reported independent effects.^8^

When well-executed and designed nutritional studies for dementia prevention obtain null results, researchers need to conclude that the null hypothesis is not disproven, rather than looking for reasons why these studies could be so-called failures. However, nearly all aspects of nutrition studies deserve scrutiny because many trials are not optimally designed (from choosing the dose, form, timing [life or disease stage], duration, target population, outcomes, and sample size)^9^ or executed. Therefore, there is an urgent need to formulate a roadmap for the design of next generation nutritional interventions for dementia prevention.

The Nutrition for Dementia Prevention Working group, formed from an international group of experts, met in 2020 and 2021 to explore these and other nutrition–cognition related issues. The aim was to guide future research by better translating findings from observational studies and experimental models into effective trials. The group gathered for a 2-day symposium sponsored by the National Institute on Aging (NIA) and the Alzheimer’s Association in June, 2021, to identify gaps and pitfalls in previous observational and interventional nutrition studies in the dementia field.

The research reviewed was organised into four major themes: (1) novel approaches for translating observational studies into the design of clinical nutrition trials; (2) precision medicine, biomarkers, and nutritional science research; (3) assessing the limitations of past clinical trials for dementia prevention; and (4) designing next generation nutritional interventions for dementia prevention. The working group concluded that nutritional interventions have unique challenges in design and execution that require both personalisation and large-scale and pragmatic study designs for more generalisable public health interventions across diverse populations. Personalisation should be guided by studying dietary networks (ie, synergistic and antagonistic connections between dietary components and nutrient biomarker patterns) and nutritional status to identify subgroups at nutritional risk for cognitive decline.^10^ Practical solutions to overcome these limitations is presented in the appendix.

## Novel approaches to translate observational studies into clinical trials: the importance of dietary patterns and nutrition assessment tools

Most randomised controlled trials of dietary interventions for cognitive health have involved nutrient and vitamin supplements. Focus on a single nutrient and a placebo control is easy to design and implement; examples previously assessed include vitamin E,^11^ vitamin D (vitamin D and omega-3 trial [known as VITAL]),^12^ and omega-3 polyunsaturated fatty acids.^13^ These straightforward experiments have led to null results (ie, findings of no association) and the null hypothesis might be true. However null results can also be caused by many other factors such as the intervention duration being too short; large proportions of the participants already having ingested sufficient quantities of the nutrient in question to be within neuroprotective range;^14^ participants being generally older than 65 years and potentially already having advanced underlying disease; and the dose being insufficient; all of which can dilute the magnitude of the effect.

### State of nutrition science from epidemiology studies

A meta-analysis of observational studies found that adherence to a healthy diet pattern is associated with a lower risk of dementia.^15^ Therefore, a diet intervention based on an individual’s dietary patterns might have more favourable effects on cognition if it alters intake of multiple foods to potentially combine many smaller effect sizes. An example of this approach is the Mediterranean Dietary Approaches to Stop Hypertension (DASH) intervention for Neurodegenerative Delay (MIND) diet, which borrows elements from a Mediterranean diet and combines foods reported to be associated with cognitive function, such as leafy greens and berries, with elements of the DASH diet, which is designed to lower hypertension. Studies of all three MIND dietary components suggest they are associated with less cognitive decline and lowered risk for Alzheimer’s dementia.^16^ The effect of the MIND diet on cognitive function is now being tested in a randomised trial^17^ and as a component of US Protect Brain Health through Lifestyle Intervention to Reduce Risk (known as POINTER) study.^18^

In addition to identifying the specific diet pattern to evaluate, researchers need to consider in whom it should be tested and when to test it (ie, age and nutritional state of the participants, and the duration of intervention). The PREDIMED (*Prevención con Dieta Mediterránea*) study, a landmark randomised controlled trial of the Mediterranean diet in Spain involved people who were young to old (median 68 years) and followed them for 5 years.^19^ A small sub-study of PREDIMED found that a Mediterranean diet supplemented with olive oil or nuts was associated with improvements in a few measures of cognition, compared with those following a control (low fat) diet.^20^

### Novel approaches to define dietary patterns using networks

If interventions are shifting toward dietary patterns, then novel approaches such as network analysis could help to provide a new understanding of the relationships between different foods and identify new dietary patterns of interest. Diet is complex, varying by time of day, week, season, environment, and culture; and network analysis can reveal non-intuitive relationships among foods (eg, non-linear). For example, a network analysis of diet data from the three-city Bordeaux study found no differences in average quantities of food intake but did find significant differences in food combinations between those with dementia and those without.^21^ With foods as nodes, and their co-consumptions connecting them, the networks of those who developed dementia were highly focused, with hubs consisting of less healthy items (eg, cured meats such as charcuterie). In contrast, those without dementia had a less connected network (ie, they ate a greater variety of foods), and more nodes centred on healthier foods, reflecting a more diverse diet. An example of using network analysis to monitor compliance to a diet is presented in the appendix (p 8).

### Evaluating time windows for dementia prevention by modelling nutrition trajectories over the life-course

Identifying the appropriate life stage for intervention is also challenging. Several modifiable risk factors are thought to be at work at specific times across the lifespan;^1^ however, the optimal times for most interventions remain unclear. In particular, the underlying process of dementia might alter lifestyle behaviours several years before diagnosis, which would make these behaviours a consequence of the disease rather than a risk factor (ie, reverse causation).^22^

The ARIC (Atherosclerosis Risk in Communities Study) showed that worsening of cardiovascular risk factors (ie, hypertension, hyperlipidemia, smoking, obesity, or type 2 diabetes) during midlife (approximately 40–50 years) had a stronger association with future dementia than these same factors in later life.^23^ Statistical modelling of lifestyle trajectories in preclinical dementia can further allow the characterisation and comparison of lifestyle behaviours between groups when combined with a nested case-control approach. This trajectory approach has been applied in prospective, observational cohorts, with lifestyle trajectories described before dementia diagnosis in the Whitehall II Study^24–26^ and the Three-City Study,^27^ and earlier cognitive decline in latelife (older than 65 years) described in the Nurses’ Health Study.^28^ In the Nurses’ Health Study, women who had substantial cognitive decline after the age of 70 years had a higher body mass index, poorer diet, and less physical activity than controls at midlife. This decline supports the belief that maintaining a healthy lifestyle in midlife might help reduce cognitive decline decades later. The challenge here is that it is not possible to do 20-year duration trials. Alternatively, biomarkers can link a dietary pattern intervention during midlife with surrogate outcomes, such as dementia risk factors. In these situations, planned lengthy follow-up studies can be used to ascertain cognitive outcomes and dementia incidence.

Overall, although we present findings from a novel trajectory method for evaluating relations of dietary risk factors for dementia during the life course, there are many different approaches that are useful. These approaches include basic age-specific analyses of risk factors and outcomes. Many different study designs and methodologies will be useful in trying to better understand the times during which interventions might be best applied to maximise health.

### Cultural approaches to consider in underrepresented groups

Another difficulty in understanding diet, either in an observational study or to gauge adherence to a diet in a trial, is capturing the diet quality among different populations, which might have their own cultural food preferences and access to different foods within the community. Typically, food frequency questionnaires (FFQs) are used to assess usual diet, in which participants answer questions about the frequency of a pre-selected set of foods and portion sizes consumed. But the foods included might not fully reflect the foods consumed by under-represented groups. Inadequate capturing of specific cultural or ethnic foods could be an important limitation and might contribute to the poor understanding of diet and cognition in studies of underrepresented minorities.^29^

With no single FFQ appropriate for all, researchers are looking for ways to improve dietary assessment tools. FFQs originally designed for non-Hispanic White people have been adapted to include ethnicity-specific foods, portion sizes, and quantities. For example, a standard FFQ was refined and validated in a study of a Puerto Rican population by adding foods like mango, green plantain, and custard flan, and by adjusting portion sizes.^30^ Web-based FFQs that use branching logic (ie, different responses to a given question lead to different subsequent questions) to adapt questions according to a participant’s answers might also capture cultural differences and food choices better.^31^ Instead of responses to a set number of food items on a FFQ, there are newer open-ended approaches with ubiquitous mobile or web-based technologies. These include repeated food recalls or records with food photography,^32,33^ household inventories, or even food purchase receipts.^34^

There has always been a need to verify subjective dietary reports using other dietary assessments with different sources of measurement error (ie, dietary biomarkers). Progress in use and development of such markers are emerging^35^ but analytical costs remain a limitation. More recently, these efforts have become more cost-efficient with the advances in omics techniques. Dietary biomarkers in blood, stool, or urine^36^ or lifestyle factors^37^ (nutrimetabolomics) in combination with traditional diet assessment has potential to improve observational studies and clinical trial design across diverse ethnic and racial populations.^38^

## Precision medicine, biomarkers, and nutrition science

Applying biomarker tools and measures to observational studies can inform the design of new trials and encourage precision medicine, in which interventions are personalised to individuals with respect to timing, dosing, and duration. These personalised tools include potential modifiers of the effect of diet on the brain such as genomics; microbiome; and biomarkers of dietary intakes, diet response (eg, endogenous metabolites), and brain ageing. The connection of the diet in relation to behavioural and systemic factors with potential modifiers is shown in figure 1.

### Defining early nutrition and metabolic signatures of disease risk by leveraging multiple candidate biomarkers or metabolomics

Biomarkers from observational studies could help to inform trial design: they can suggest the dosage and duration of an intervention, help to estimate the power and required sample size, and define which participants are perhaps most sensitive to the effect of intervention and would be optimal for inclusion in clinical trials (appendix pp 3–10).

Biomarkers in cohorts could also be used to screen a population sensitive to nutrition intervention for trial eligibility, potentially focusing on those with poor nutritional status. For example, a nutritional risk index that combined nutrient biomarkers of omega-3 fatty acids, homocysteine, and vitamin D was associated with cognitive decline in a secondary analysis of the large Multi-domain Alzheimer’s Prevention Trial (MAPT).^10^ A similar index used in the three-City study was also associated with a higher risk of dementia, with a large effect size.^39,40^ Effect sizes can be used to estimate sample size (appendix p 10). Importantly, the magnitude of reported associations in the three-city study was high (ie, stronger than the effect size of *APOE* ε4 status). This finding suggests that establishing neuroprotective thresholds for nutrients and treating any insufficiencies in multiple nutrients with a multinutrient diet, dietary pattern, and supplement interventions might provide a better signal of nutrition effect.

Several other tools to examine the state of different molecular pathways, such as transcriptomics, metabolomic, genomics, and the gut microbiome will probably help to capture the body’s response to dietary intake. The heterogeneity in the response to dietary bioactives from food intake is a tenet of personalised nutrition.^41^ The use of these tools could elucidate which pathways are altered during early dementia that might be correctable by appropriate diet, facilitating the identification of key risk profiles within a personalised medicine framework. The tools might also help to refine the characterisation of optimal nutrient or food combinations and reveal potential novel therapeutic nutrition. Precision biomarkers are key to the design of prevention trials tailored to an individual’s biological and nutritional status.

### Interplay between genetic background and nutritional metabolism on dementia risk

Genome wide association studies have substantially contributed to our understanding of dementia and Alzheimer’s disease, through identification of novel genetic risk loci. There is anticipation that the combination of genetics and nutrition research can begin a new phase of personalised medicine and personalised health in the treatment and prevention of dementia.

Genetic studies can provide insights into underlying mechanisms and gene–nutrient and gene–diet interactions. This knowledge can then be used both to identify the potential preventive utility of lowering dementia risk through nutrition in people with genetic risk and to improve future clinical trial designs, for instance through recruitment of populations with certain polymorphisms. Beyond traditional genetic studies, the emergence of novel omics technologies (eg, epigenetics and metabolomics) provides further opportunity to disentangle the biological effects of diet and nutrition on the brain and the manifestation of genetics on whole systems. Studies point to the possibility that a healthy lifestyle might offset some genetic risk for dementia,^42^ except in the presence of a high genetic burden.^43^

### The link between nutrition and brain health through study of microbiota

The intestinal microbiome might mediate (and potentially moderate) some responses to diet. Although the basic composition of bacterial species in the intestine is largely the same across people, individuals have differences that result in varied metabolic responses to the same diet.^44^ Underlying dietary patterns can influence the capacity of the gut microbiome to produce certain metabolites; for example, one study of omnivores and vegans found that omnivores produce significantly more trimethylamine-N-oxide, an atherosclerosis-promoting metabolite, after eating a protein-rich meal.^45^ Gut microbiota are required to form trimethylamine-N-oxide and several bacterial taxa were significantly more abundant in omnivores than in vegans (eg, *Peptostreptococcaceae incertae sedis, Clostridiaceae, Peptostreptococcaceae, Clostridium*), which could affect an omnivores ability to synthesise trimethylamine-N-oxide.^45^ Similarly, a study of the Mediterranean diet and cardiovascular risk found that people with *Prevotella copri* in the microbiome did not benefit from the diet, whereas those without *P copri* had a substantial decrease in risk for myocardial infarction.^46^ These studies illustrate the need for precision medicine based on information about an individual’s genetics, omics-based biomarkers, and gut microbiome.

Efforts are ongoing to understand how the diet and intestinal microbiome affect the brain. The Alzheimer’s Gut Microbiome Project is combining multiple nutritional trials including the MIND trial,^47^ the BEAT-AD trial of a modified ketogenic diet (NCT03472664), and the US POINTER trial.^18^ Detailed analysis of microbiota composition and function in these studies will be used to derive a comprehensive and mechanistic understanding of diet–microbiome–cognition–brain structure associations. The identification of key relationships between dietary intervention, specific gut microbiota, and cognition is essential as they will facilitate the testing of new hypotheses that might lead to new treatments. Additional examples of the utility of gut microbiome applications in nutrition clinical trials can be found in the appendix (p 6).

### How brain imaging can guide the efficacy of nutritional interventions

Neuroimaging methods have evolved since the early 2000s, giving researchers access to many features of the brain that associate with risk or resilience to cognitive decline. With trials for Alzheimer’s disease moving to intervene before disease onset, the use of neuroimaging offers alternative outcomes (ie, surrogates) that precede changes detected clinically on neuropsychological testing. Such biomarkers could also be used to characterise participants for stratification into subgroups or to identify their eligibility for a study.^48^ Brain imaging measures are numerous and diverse, ranging from structural measures of brain volume and cortical thickness, or white matter lesions and integrity, to cerebrovascular metrics (ie, infarcts) and functional MRI that captures brain metabolism, such as PET, which can detect the amyloid or tau burden, markers of metabolism (eg, glucose), and inflammation.

Many studies have examined links between diets and brain measures, but the results have been inconsistent. Given that these studies are small, make use of different imaging methods, and are mostly cross-sectional in design, more studies are needed to clarify the role of diet in brain imaging measures. Incorporating brain measures in diet trials is also important because examining imaging surrogates of brain health can help to shorten trial duration and identify susceptible or sensitive populations for a trial. For example, the LipiDiDiet trial found beneficial effects for Nutricia Souvenaid (a supplement for dietary management of Alzheimer’s disease) on reduced hippocampal atrophy at 24 months,^49,50^ but significant effects on the primary cognition outcome could only be seen at 36 months.^51^ In the Finnish Geriatric Intervention Study to Prevent Cognitive Impairment and Disability (FINGER) trial, the multidomain intervention, which included a dietary component, was more effective for cognition among patients with more intact brain morphological measures (ie, baseline cortical thickness or hippocampal volume).^52^ Although diet interventional studies are ideal, observational studies could also provide crucial information. For example, observational studies can provide an estimation of the typical trajectory over time of brain measures (ie, longitudinal study) or age-related changes (ie, cross-sectional studies), which will help to identify the appropriate duration for a future trial. More granular voxel-wise analysis from observational studies can identify brain regions that are closely linked to a specific cognitive test, as done in 2020 with MAPT participants.^53^ Imaging might also give mechanistic explanations for associations between diet and cognition. For example, white matter tract integrity might be responsible for the cognitive benefits of a healthy dietary pattern^54^ and omega-3 fatty acids,^55^ whereas brain atrophy, as measured by decreased grey matter volume, is a potential mechanism explaining the association between an inflammation-promoting diet and worse visuospatial cognition.^56^

## Limitations of past nutrition and supplement clinical trials for dementia prevention

Three recent randomised controlled trials of multi-domain interventions that included a dietary component found no or only small effects on cognition (table). The paucity of strong results raises questions about whether trial designs were adequate to identify large effects if they exist. Knowledge from these trials underscores the importance of many factors for nutrition and dementia prevention such as the intervention intensity, treatment, and follow-up duration; adherence; baseline population characteristics including cognitive status, future dementia risk, levels of vascular comorbidities, education, or other cognitive reserve-related variables; adequate sample size; and chosen outcomes. It appears that prevention trials need to aim an optimum balance of finding people who are at risk for dementia, but who have not already developed the disease. These trials might also require more intensive nutritional interventions and a longer length of intervention or follow-up. Some methodological aspects can be partly remedied with pragmatic study designs (appendix pp 9–13).

### Limitations of the major nutrition and multidomain trials for prevention of cognitive decline

The FINGER trial^5^ in Finland involved a two-year multidomain (diet, exercise, cognitive training, and vascular risk monitoring) intervention compared with a control group that received general health advice. The trial found that an improvement of diet, exercise, and cognitive stimulation had beneficial effects on cognition in terms of executive function and processing speed compared to those in the control condition. The use of sensitive cognitive outcomes; the age range of the selected population and increased cognitive decline risk; and the intensive nature of the intervention and the high compliance and adherence might have been the keys to its success.

The Prevention of Dementia by Intensive Vascular Care (preDIVA) trial in the Netherlands was a pragmatic study that aimed to prevent dementia through a multidomain intervention (three visits a year in which a practice nurse addressed vascular risk factors with medical and non-medical interventions) meant to reduce cardiovascular risk over the course of 6 years. The trial did not detect an overall benefit on the primary outcome all-cause dementia.^7^ PreDIVA post-hoc subgroup analyses suggests that those with untreated hypertension who were adherent to the intervention (around 28%) showed a reduction in dementia risk. Extended observational follow-up of up to 12 years did not show a delayed effect.^57^ In PreDIVA, the population might have been older than ideal for the intervention, the intervention might not have been sufficiently intense, and the outcome, although clinically meaningful and pragmatic, might have been insensitive to some of the intervention effects.

The MAPT trial in France was a multidomain intervention (physical activity, cognitive training, and nutritional advice) that was combined with or without omega-3 supplements in a very broad population. After 3 years, no cognitive benefits were apparent.^6^ Participant selection might have played an important role in MAPT’s results as the study’s population was older (mean 75·3 years) with elements of frailty susceptibility, but of higher cognitive reserve and of lower future dementia risk. Furthermore, the nutritional intervention could have had better content and greater intensity. Such an intervention might show an effect in a more targeted population, such as in *APOE* ε4 carriers, or in those with cerebral amyloidosis, high dementia risk, or poorer baseline nutrition.^58^

### Personalised diets based on dietary patterns

A whole diet based on dietary pattern intervention might produce meaningful biological effects, but there are few of these trials. These studies are difficult to implement and, if there is an effect, it is difficult to identify the components of the diet responsible for any beneficial outcomes. Also, for whole diet studies it is difficult to establish what the optimal control diet should be. An ambitious feeding trial of the MIND diet (NCT02817074) is ongoing, aimed at preventing Alzheimer’s disease in an at-risk population (ie, those with obesity, a family history of dementia, and a suboptimal diet). The trial’s primary outcome is a change in global cognitive composite score with additional surrogate outcomes, including brain volume and measures of cardiovascular health and metabolism. In general, biomarkers for Alzheimer’s disease such as plasma or CSF amyloid-beta and tau could also be monitored.^59,60^

The ketogenic diet is low in carbohydrates and high in fat—a combination that increases the production of ketones, which have neuroprotective effects. Although it is a substantial overhaul of a typical diet, previous small studies suggest that following a ketogenic diet for a short duration can improve memory scores for people with mild cognitive impairment.^61,62^ One other pilot ketogenic diet trials that assesses brain outcomes is in progress now: a four-month trial of the Modified Mediterranean Ketogenic Diet (MMKD; NCT03472664) in 120 people with mild cognitive impairment. In 2019, a six-week trial of MMKD^63^ resulted in improvements of Alzheimer’s disease-related biomarkers and changes to the gut microbiome. There were no adverse effects, but the diet might be most feasible if prepared meals are provided to participants.

Feeding trials might remedy many of the feasibility challenges (including participant training and researcher assistance of participants meal preparations, and variability of adherences to nutritional intervention) of more traditional dietary interventions and socioeconomic biases but are more costly and challenging in other respects.

Larger trials will be necessary to obtain reliable results, especially if there are differences in individual responsiveness to the diet. Given the variability of individual responses to the same diet, use of measures like post-prandial glycaemic response^60^ could help to tailor new interventions. Overall, integration of nutrition science towards more personalised individual responses and multidimensional data-analytical approaches could be considered.

### Supplements and cognition trials

Non-prescription supplements are commonly marketed with claims about improving memory or brain function and might be consumed with the intent to prevent dementia. Although trials of supplements are easier to implement than food-based diet trials, and many supplement trials have been done, no consistent benefits have been detected. In general, most trials have a suboptimal design: a 2017 review^64^ reported that many trials had a high risk of bias, among trials with low to medium risk of bias common problems included low supplement dosage; insufficient information about participants’ baseline nutrient intake levels; a paucity of baseline cognition measures; high attrition rates; small sample sizes; short durations; and paucity of objective measures of adherence throughout the trial.^64^ Furthermore, participants’ baseline nutritional status was infrequently known, raising the risk that a high proportion had already sufficient nutritional status of the nutrients under study. These issues combined limit the ability to detect cognitive changes in past trials but might be instructive for future trial design. We need well-designed and executed trials in participants at midlife and in populations with suboptimum nutrient intakes that have been associated with risk for cognitive decline. Standardisation of dose, trial populations, cognitive outcomes, and methodological approach in general could help to build the trial data that could then be pooled across studies to obtain conclusive results to inform public health.

## Designing nutrition clinical trials for dementia prevention

Study designs leveraging biomarkers, genetics, and other tools in the clinical trial setting can help to translate specific observational studies into effective clinical interventions. However, the nature of nutrition-based studies requires specific trial designs (appendix pp 9–13) to accommodate the complexity of these interventions, such as at what point during the life course or disease stage they need to be applied, and the intervention duration. No single diet will fit the preferences of all participants, which argues for a more nuanced approach to whole diet studies. Nutritionists can help to match participant preferences with a diet’s variables, which can help with study recruitment, adherence, and retention of diverse groups of participants. As studies shift toward using biomarkers either as participant selection variables or as surrogate outcomes in these studies, it might be worth considering these as Phase 2 proof-of-concept studies, rather than the randomised controlled phase 3 efficacy trials.

### Experience in the execution of prevention nutrition–cognition trials: practical problems and challenges

There are several practical challenges in doing nutrition and cognition trials. Many problems revolve around time: not only the long time it takes to see an effect from a lifestyle intervention, but also the time it takes to plan and recruit for a large trial. These types of trials tend to be multiyear endeavours, during which the knowledge and best practices can evolve, prompting changes in assessment tools and changes in primary outcome. For example, during the MAPT trial^6^ new cognitive outcomes were developed, which were adapted, leading to changes in the primary outcomes. For the long time it takes to observe efficacy, one solution could be multiarm, multistage adaptive trials in which several interventions are compared to one control arm. Similarly, when designing a study, power analyses to determine sample size, despite being based on the most current data, might be outdated by the time a study begins. An example of this scenario came from the GuidAge trial,^65^ in which educational attainment in the target population increased by the time the study began. This increase is associated with less cognitive decline, making it harder to see an effect. A potential solution to this issue might be data sharing between ongoing trials through the publication of study design and baseline characteristics at a study’s outset.

Access to a target population is always complicated and might require considerations of incentives for general practitioners in primary or preventive health-care systems to recruit participant pools. Competing recruitment between academia and pharmaceutical companies can also be a challenge if a diet intervention appears to be less innovative than a drug to potential participants. Also, participants might not necessarily understand the concept of a prevention trial and its requirements: the importance of adhering to the diet, that it will take an extended period of time, that one must stay in the assigned trial arm, or even that researchers are looking for a link between nutrition and cognition.^9^

**Figure 2:**
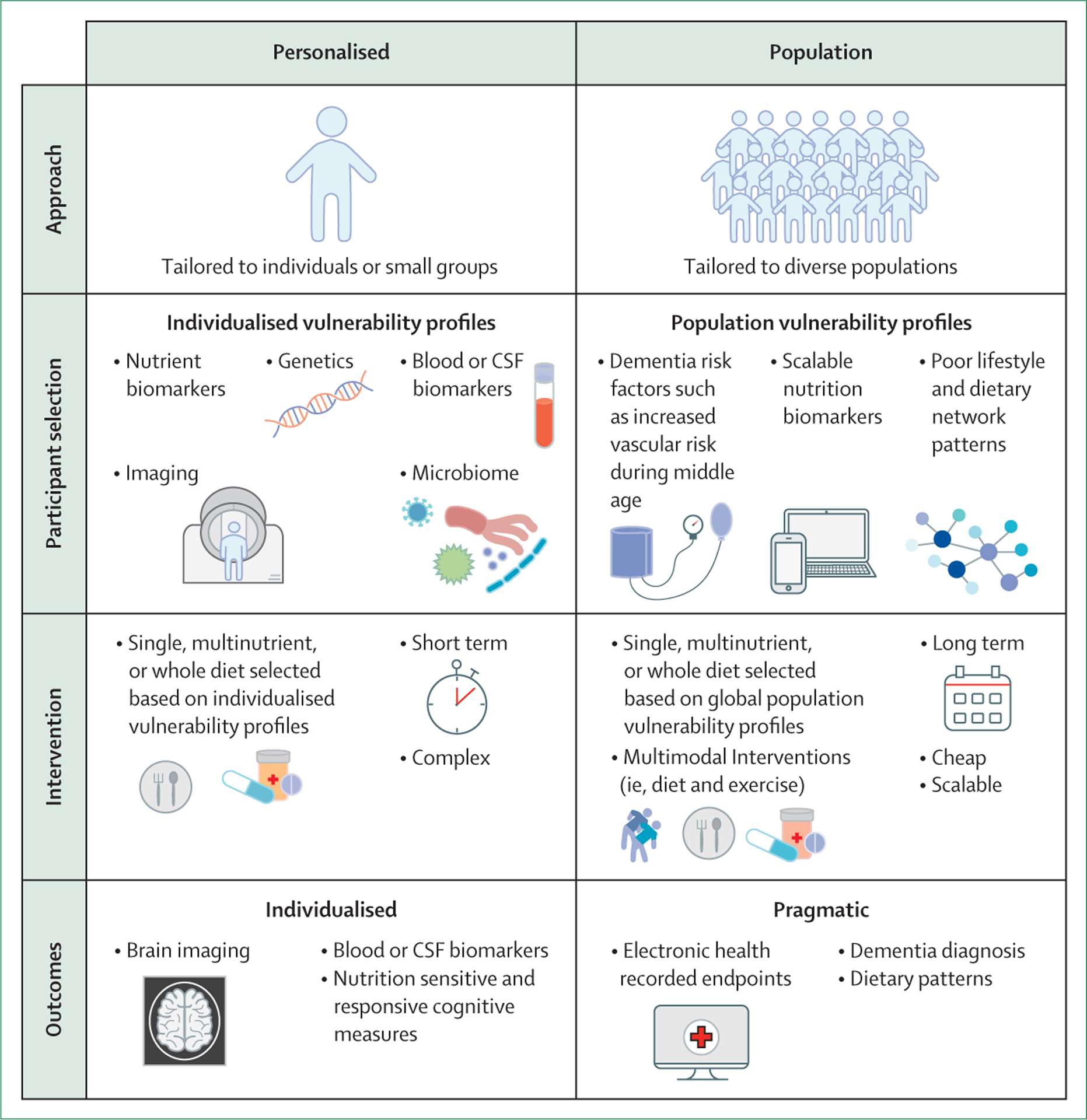
Two contrasting study designs to nutrition-based interventions for dementia prevention Two contrasting approaches to nutrition-based clinical trials are shown. The first column shows intensive and personalised interventions guided by biomarkers that capture brain functions. In the second column, interventions are tailored to a population level in groups at risk of dementia and uses pragmatic outcomes. Although certain trials will have to share elements from both approaches, clear study designs that match the intensity of the intervention with the outcome proposed promises to maximise the chances of finding effective therapies.

New ideas for the next generation of interventional studies include phase 2 precision medicine trials by use of biomarker-based outcomes, trials of longer duration, and larger preventive trials, with primary outcomes assessing change in function or activities of daily living, rather than using cognitive tests as a proxy of functional decline (figure 2). Larger scale preventive trials might make use of biomarkers for a full sample (rather than just a subgroup), with centralised analyses for both accuracy and cost reduction, but not as an outcome measure. A typical randomised controlled trial might never fully test whether findings from observational studies are causal or not, unless there is careful attention to personalising the intervention. By contrast, an adaptive trial design allows investigators to adapt the intervention based on initial response in the study population. Several trial designs can be used by researchers to overcome some of the past limitations in nutrition-based interventions (appendix pp 11–12).

### Designing small scale personalised trials

Developing and designing more targeted and biomarker-based nutritional interventions (ie, precision nutrition trials) can offer a rigorous way to assure that an intervention reaches the target thresholds of dietary nutrients or related metabolites, and that the primary outcomes are sensitive to the interventions. Knowledge of a proposed nutritional intervention’s pharmacokinetics and pharmacodynamics can help assure that the proposed nutritional intervention reaches a therapeutic range hypothesised to be neuroprotective on the basis of other observational or interventional studies. Such interventions can start with very small but well-defined groups^48^ to test whether they appear to engage the target mechanisms and outcomes, which were hypothesised to be sensitive to the intervention in individuals at risk for dementia (ie, vulnerable populations). Various clinical trial designs can be considered (appendix p 6).

Nutrition’s effects on cognitive health might be amplified in specific population subgroups (ie, those with suboptimal nutritional status, *APOE* genotypes), requiring a personalised approach. One example is the *APOE* ε4 that carries the strongest genetic risk for late onset Alzheimer’s disease in some populations. *APOE* ε4 is associated with the cellular metabolism of lipids and glucose, and might affect how weight loss, exercise, and diet affect cognitive risk.^66^ The use of omega-3 fatty acids by those who carry *APOE* ε4 appears to differ by age, sex, and disease stage compared with those who do not,^67^ with evidence from epidemiological studies suggesting that those with the *APOE* ε4 allele might require an increased omega-3 intake at a younger age.^68^ Here, a precision medicine primary-prevention approach based on genetics, age, or dietary habits guided by brain biomarkers could have a major role in designing a nutrition-based intervention for preserving cognitive functions and delaying disease onset decades before decline.

### Designing large scale interventions

Taking a public health perspective, multidomain interventions will need to be affordable and scalable to reach enough people to make an impact at a population level. As for any successful trial, the intervention will need to reach the right people, at the right time, in the right way. Finding the optimal age for intervention is difficult because dementia emerges later in life, despite the risk factors and neuropathological processes that lead to dementia being active in midlife.^69^ The target population also needs careful consideration: if an intervention is focused on a few people at high risk for dementia, then it will have a low population effect; whereas if a study targets people at intermediate risk (eg, defined by simple measures of risk based on demographics or family history), then there will be greater potential to positively affect more people at the population level, even if the effect at the individual level is small.

To find effects at the level of public health, trials need to go large and be pragmatic. Scalable electronic-health interventions that are web-based are feasible for people older than 65 years,^70^ although personal interaction or coaching is essential to connect with participants for motivation and adherence to a lifestyle intervention. Similar interventions using mobile phones, which are particularly well-suited to low-income and middle-income countries, are also being developed (eg, Prevention of Dementia using Mobile phone applications [PRODEMOS]; a blended coach-supported health intervention in which participants can improve their dementia risk factors using a mobile health app to set and monitor goals).^71^ Mobile phones might eventually deliver cognitive tests to participants, which could make them collectors of outcome data in large trials.

One consideration for pragmatic trials is that the diet’s effects on cognition could be indirect and might take decades to affect dementia incidence, working through the reduction of other risk factors such as obesity, diabetes, and cardiovascular disease. Planning for this association requires an understanding of how dietary patterns interact with dementia risk factors to increase cognitive decline and targeting these patterns with pragmatic, large-scale, and multimodal interventions. Future dietary interventions might have immediate outcomes that could focus on dementia risk factor reduction as opposed to cognition and dementia incidence. More information on study designs is presented in the appendix (pp 9–11).

### Consideration of dementia prevention clinical trials in underrepresented groups

Although Alzheimer’s disease disproportionately affects minority racial and ethnic groups, and socioeconomically disadvantaged people, these groups are underrepresented in clinical trials. Finding ways to recruit these underrepresented groups is essential because they could benefit the most from these interventions, and their inclusion might result in bigger effect sizes. Also, trials comprised of participants more representative of the population might be more generalisable. Barriers to inclusion of underrepresented groups include poor proximity to academic centres, potential language barriers, digital divide (eg, connectivity and network issues, and comfort with technology), work schedules, and transportation access that limit attendance for frequent study appointments. A longstanding established distrust in research by some groups is also a key barrier. To encourage the participation of underrepresented minorities in Alzheimer’s disease research, a recent meta-analysis highlighted the importance of community outreach, the need to establish trust, offers of financial compensation or transportation, and meeting in a familiar location.^72^ Challenges and solutions of doing trials in underrepresented groups is discussed in the appendix (p 13).

## Conclusions

In conclusion, we recommend a roadmap (panel) for future nutrition clinical trials for dementia prevention that makes use of two different approaches, with unique goals and study designs. One approach that is intensive, personalised, and guided by patterns, network analysis, hypothesis-driven diets, and biomarkers, and another approach that is more scalable and pragmatic at a population level but might use either intermediate (biomarker) or hard clinical endpoints such as developing dementia (figure 2). In both approaches, addressing diversity and cultural dietary preferences is important. In the shift toward the use of biomarkers in smaller personalised trials, there are still questions about how surrogate biomarkers of future cognitive decline translate into real world clinical benefits. The field needs to establish the biomarker thresholds of nutritional metabolism associated with neuroprotection, and whether any of these biomarkers correlate with treatment improvements in clinical outcomes. Retooling analyses of standard measurements might help to find outcomes that reflect real improvements, such as specifying what a clinically important change in a standard measure would be.^73^ Researchers could then calculate the proportion of participants who have clinically important changes, which might help to detect subgroups that would benefit from an intervention. Pragmatic scalable trials of nutrition and other lifestyle interventions targeting those with dementia risk factors might be most beneficial at midlife but are often judged as being too short. Identifying the ideal length of the intervention is difficult but should depend on the hypothesis about how the intervention works. One intermediate approach might be to look for a risk factor reduction on a short time scale, and then follow up with participants many years later to see if the risk factor reduction made a difference in clinically important outcomes. The working group recommends against repeating trials with difficult-to-implement dietary interventions, targeting heterogenous and nutrient replete groups of individuals, using cognitive outcomes that do not reflect how the diet affects the brain, or trial durations that preclude sufficient follow-up to detect potential effects on cognition or dementia outcomes.

## Supplementary Material

1

## Figures and Tables

**Table:** Summary of three major multidomain interventions for dementia prevention

	PREDIVA^7^	FINGER^5^	MAPT^6^
Participant age (years)	70–78	60–77	≥70
Sample size	3526	1260	1680
Intervention	1890 in the multidomain cardiovascular intervention; 1636 in the control group (usual care)	631 in the multi-domain intervention; 629 in the control group (general health advice)	420 in the multi-domain intervention with placebo; 417 in the multi-domain intervention with omega-3 polyunsaturated fatty acids; 423 in the omega-3 polyunsaturated fatty acid alone group; 420 in the placebo alone group
Original duration (years)	6–8	2	3
Outcome	Clinically assessed; dementia incidence; disability score	Neuropsychological test battery Z score	Z score combining 4 cognitive tests; disability score; frailty score
Comments	The study had a population-based sample that was not selected for dementia risk, a large sample size, a long duration, and a representative population with average dementia risk; it was a low intensity intervention with an insensitive but clinically relevant outcome	The study had a population with high dementia risk, a small sample size, and a short duration; the outcome was sensitive, and the intervention intense	The study had a large sample size, a long duration, and a heterogeneous population (higher reserve, low vascular and low dementia risk); the outcomes were sensitive; the nutrition intervention could have been of better content and intensity

FINGER=Finnish Geriatric Intervention Study to Prevent Cognitive Impairment and Disability. MAPT=Multi-domain Alzheimer’s Prevention Trial. PREDIVA=Prevention of Dementia by Intensive Vascular care.

## Panel: A roadmap for future studies in nutrition and dementia prevention

### Theme 1: novel approaches to translate observational studies

- Expand on the small number of identified diet patterns
- Use network analysis to understand patterns in diverse cultures
- Improve diet assessment tools for diverse populations
- Identify the optimal age for dietary interventions to reduce adverse cognitive outcomes

### Theme 2: precision medicine, biomarkers, and nutrition science

- Identify and validate biomarkers of nutritional metabolism and thresholds associated with neuroprotection to guide clinical trial design
- Standardise and harmonise some research tools (eg, omics or brain imaging-based) across research groups
- Develop and validate blood and imaging biomarkers as surrogate outcomes that capture suboptimal nutritional intake and response to nutrition interventions, particularly during midlife
- Build consortia to increase sample sizes and allow the study of gene-by-nutrition interactions
- Incorporate the study of microbiota into both observational and interventional studies to understand the response to the diet

### Theme 3: lessons learned from past trials

- Target populations or groups with dementia risk factors before the onset of dementia, as opposed to the general population
- Test supplements in populations with low or insufficient nutrient intake, at midlife, and use validated brain-related outcomes reflective of how these supplements affect the brain
- Measure responsive biomarker outcomes during the trial (eg, post prandial glucose or ketone bodies)

### Theme 4: new trial designs

- Define the comparator or control groups using dietary pattern network analysis before the start of the trial
- Design whole diets or medical foods based on multiple neuroprotective dietary or nutrient components that can be applied in interventional trials
- Design smaller personalised trials that consider genetics, omics, microbiome, and nutrient exposures guided by biomarkers that reflect brain functions
- Design larger pragmatic electronic-health trials targeting populations with dementia risk factors and focusing on reducing dementia risk factors
- Adaptive randomised trials with protracted run-in periods might allow researchers to better monitor compliance with control diets before randomisation
- Cluster randomised trials randomisation (eg, study sites as opposed to individuals) would probably increase compliance with the intervention within the site
