# Supplementary material for: Nutrition state of science and dementia prevention: recommendations of the Nutrition for Dementia Prevention Working Group
Source: Lancet Healthy Longev. Author manuscript; Available in PMC 2022 Jul 11. (PMC9273104; doi:10.1016/s2666-7568(22)00120-9)
Supplement: 1 [file NIHMS1821266-supplement-1.pdf]

THE LANCET
Healthy Longevity

Supplementary appendix

This appendix formed part of the original submission and has been peer reviewed. We post it as supplied by the authors.

Supplement to: Yassine HN, Samieri C, Livingston G, et al. Nutrition state of science and dementia prevention: recommendations of the Nutrition for Dementia Prevention Working Group. *Lancet Healthy Longev* 2022; **3:** e501–12.

Nutrition State of Science and Dementia Prevention: Recommendations of the Nutrition for Dementia Prevention Working Group

APPENDIX

Hussein N. Yassine*, MD (Departments of Medicine and Neurology, Keck School of Medicine, University of Southern California)
Cécilia Samieri*, PhD (Univ. Bordeaux, INSERM, BPH, U1219, F-33000 Bordeaux, France)
Gill Livingston, MD (Division of Psychiatry, University College London and Camden and Islington NHS Foundation Trust.)
Kimberly Glass, PhD (Channing Division of Network Medicine, Brigham and Women's Hospital. Department of Medicine, Harvard Medical School. Department of Biostatistics, Harvard Chan School of Public Health)
Maude Wagner, PhD (Rush Alzheimer's Disease Center, Rush University Medical Center, Chicago, IL, USA. Department of Neurological Sciences, Rush Medical College, Chicago, IL, USA.)
Christy Tangney, PhD (Departments of Clinical Nutrition and Preventive Medicine, Rush University Medical Center)
Brenda L. Plassman, PhD (Department of Psychiatry and Behavioral Sciences Duke University Durham, NC USA)
M. Arfan Ikram, MD, PhD (Department of Epidemiology, Erasmus MC University Medical Center, Rotterdam, the Netherlands)
Robin M. Voigt, PhD (Rush Center for Microbiome and Chronobiology Research, Rush University Medical Center, Chicago IL. Department of Internal Medicine, Rush University Medical Center, Chicago, IL. Department of Anatomy and Cell Biology, Rush University Medical Center, Chicago, IL)
Yian Gu, MD PhD (Departments of Neurology and Epidemiology, Taub Institute, and Sergievsky Center, Columbia University Irving Medical Center, New York, NY.)
Sid O'Bryant, PhD (University of North Texas Health Science Center)
Anne Marie Minihane, PhD (Norwich Medical School and Norwich Institute of Healthy Ageing, University of East Anglia, Norwich, Norfolk, UK)
Suzanne Craft, PhD (Department of Internal Medicine-Geriatrics, Wake Forest School of Medicine)
Howard A. Fink, MD, MPH (Geriatric Research Education and Clinical Center, Minneapolis VA Health Care System, Minneapolis, MN, USA.)
Suzanne Judd, PhD, MPH (University of Alabama at Birmingham, Biostatistics School of Public Health)
Sandrine Andrieu, MD, PhD (Aging Research team, CERPOP, Inserm, Université Paul Sabatier, and Clinical Epidemiology and Public Health Department, CHU de Toulouse, France)
Gene L. Bowman, ND, MPH (Oregon Health & Science University, Department of Neurology, NIA-Layton Aging and Alzheimer's Disease Research Center and National University of Natural Medicine, Helfgott Research Institute, Portland, OR, USA)
Edo Richard, MD, PhD (Department of Neurology, Donders Institute from Brain, Behavior and Cognition, Radboud University Medical Centre, Nijmegen, The Netherlands and Department of Public and Occupational Health, Amsterdam University Medical Centre, location University of Amsterdam, The Netherlands)
Benedict Albensi, PhD (Nova Southeastern Univ. - College of Pharmacy, Dept of Pharmaceutical Sciences, USA. St Boniface Res Ctr and the Univ of Manitoba-Department of Pharmacology & Therapeutics, Canada)
Emily Meyers, PhD (Alzheimer's Association)
Serly Khosravian (University of Southern California)
Michele Solis, PhD (Alzheimer's Association)
Maria Carrillo PhD (Alzheimer's Association)

Heather Snyder, PhD (Alzheimer’s Association)
Francine Grodstein*, ScD (Rush Alzheimer's Disease Center, Rush University Medical Center, Chicago)
Nikolaos Scarmeas*, MD (Department of Neurology at Aiginitio Hospital, Medical School, National and Kapodistrian University of Athens, Athens, Greece; Department of Neurology, Columbia University, New York NY, USA.)
Lon S. Schneider*, MD, MS (Della Martin Chair of Psychiatry and Neuroscience, Professor of psychiatry, neurology, and gerontology, Keck School of Medicine of USC)

* Program committee, equal contributions

Introduction

The Nutrition for Dementia Prevention working group presented a summary of the Nutrition State of Science Symposium at the Alzheimer's Association International Conference in July of 2021. The presentation was followed by a discussion of the limitations and barriers to progress in this field. In this appendix, we summarize these discussions and provide practical solutions for a future roadmap. The topics covered included participant selection, biomarkers, testing a hypothesized mechanism, defining the comparator group and compliance, aspects specific to supplements, novel trial platforms and issues pertinent to research in underserved areas. The working group recommendation for a roadmap to guide nutrition-based interventions for dementia prevention is provided at the end of this document.

1. Participant selection: Possible limitations of previous trials include enrollment of participants who already consume a healthy diet may reduce a study's ability of a healthy diet intervention to induce benefits. Because study participant heterogeneity in existing diet in previous trials may have thwarted finding benefits for cognition, future trials may need to consider selection of participants based on baseline characteristics relevant to the intervention under study (although this adds complexity and expense to study recruitment). Studies will have to identify and enroll participants whose dementia risk is modest, as well as recruit those who may most benefit from an intervention. This could mean enrolling people with suboptimal nutrition, or those who have low intake levels, though optimal nutrient and food thresholds have not yet been established for cognitive health. Additional consideration is warranted for those with unhealthy lifestyles, who not only have higher risks for future cognitive decline but who may also live further from research centers and require additional effort for recruitment. Future studies may improve upon past studies in a number of ways. Improved estimates of nutritional exposure, including a comprehensive appraisal of the food metabolome or network analyses of food patterns, could

better inform interventions. Personalized approaches that consider a person's genetics or biomarker status may help select participants for a study, as opposed to a post-hoc analysis of existing sub-cohorts.

2. Biomarkers: For improving participant selection into clinical trials, the use of biomarkers is critical. We discuss here how biomarkers can be used to advance trials and key areas that require additional research.

a) Blood biomarkers: Blood biomarkers can be used as surrogate endpoints for smaller personalized phase II clinical trials, or in larger prevention trials to select a population with low levels of this biomarker, and to track the effect of supplementation. Blood biomarkers of AD, such as amyloid or pTau, could also be employed to enroll high risk groups but only if the diet components are known to affect these AD biomarkers. It remains to be determined whether a change in these markers reflects a potential benefit for long-term cognition. Other types of biomarkers emerging from the "–omics" fields await validation before they are ready for clinical trial use. Biomarkers may also eventually provide objective measures of diet adherence, but validation for this purpose requires further study. Combinations of measures or biomarkers may also provide useful composite endpoints for phase II trials. The field is in early stages of biomarker use, and collaborations may help expedite their use by establishing standards and protocols to validate their use.

As potential biomarkers multiply, the field is at the very beginning of validating their use. Methods across the many domains (genetic, transcriptomic, epigenomic, metagenomic, metabolomics, proteomic, brain imaging-based, etc.) need to be standardized across research groups to provide robust and replicable answers on what these biomarkers signal, and how they may be used in

studies. Though finding biomarkers that indicate something about diet or cognitive health would be helpful, it is also important to consider that these measures may differ between different ethnic or racial subpopulations. Findings in observational studies need to be followed up and validated on a large scale to advance precision medicine. There is also a tension between precision medicine and scalability: as more complicated measures are used to precisely index the nutritional or cognitive status of an individual (e.g., multilayer omics), the approach becomes more difficult to both replicate and implement in a clinic and on a population level. A trade-off between accuracy and simplicity should therefore be sought.

One difficulty for nutrition trials is in defining both nutrient intake levels needed and circulating nutrient levels to reach for optimal cognitive health. While classical nutritional deficiencies have been established, the defined thresholds may not hold true for brain health. For example, vitamin D levels needed for bone health may be lower than that needed for an anti-inflammatory action conducive to brain health. No threshold of any nutrient has been calibrated to cognitive function.

b) Brain Imaging biomarkers: Brain imaging offers many pre-clinical endpoints, ranging from structural and vascular markers to measures of functional brain activity or PET-detected signs of pathology and neurodegeneration. If these measures detect meaningful changes in brain health before cognitive changes are apparent, they might help shorten trial duration. Future research of brain imaging biomarkers and nutrition should focus on prospective designs, cohort collaborations, multi-modal analyses, and standardized collection protocols.

c) Genetic biomarkers: To leverage opportunities of integrating genetic biomarkers into clinical trials, several challenges also need to be met. These include issues revolving around power and sample size, sufficient replication of findings, and proper interpretation of findings and synthesis

into complex biological systems. Finally, there is a challenge in translating such complex biological knowledge into simple language that is understandable (and motivational!) for the general population.

d) The application of the gut microbiome to nutrition trials: There are many approaches that could be tested once we clearly understand the underlying relationship between the gut microbiota and cognition in large populations of diverse individuals. Microbiota modulation approaches such as prebiotic, probiotics, or synbiotics could be used to increase or decrease the abundance of specific bacteria or bacterial groups (i.e., clades) that influence cognition. The use of bacteriophages could be investigated as a new treatment approach to remove bacteria that are detrimentally associated with cognitive impairment and dementia, including AD. If bacterial function (e.g., metabolites) proves to be more important than the abundances of specific bacteria, then an approach that modifies bacterial function could be used. An example of one such approach is targeting bacterial CutC/D enzymes to limit the production of TMAO which is implicated in cardiovascular disease(1). Challenges that remain, include standardizing collection of microbiome samples, technical issues like the impact of long-term storage and analyses techniques, as well as biological issues like sample site collection (stool versus mucosal-associated microbiome) and timing of sample collection (i.e., circadian rhythms).

3. Testing a hypothesized mechanism: Outcomes in a trial should reflect a hypothesized mechanism of action, such as vascular, inflammatory, or neurodegeneration-related signs in the brain and possibly distinguish cognitive aging from dementia. As a rule, clinical trials need to be based on preliminary data that provide a strong hypothesis for how the intervention works. Specific diet patterns, such as a Mediterranean diet, show strong effects on cognition in observational studies, and deserve to be followed up further with careful hypothesis driven

interventions. A future Mediterranean diet study might begin with a focused, small pilot study that monitors blood and neuroimaging biomarkers based on known mechanism of protection (from preclinical studies) as an outcome, in people with a less "Mediterranean" nutritional status. A second example is an intervention that aims to reduce insulin resistance might improve vascular biology, which in turn maintains brain health, and lowers risk for cognitive decline. Such intervention may not work through amyloid or pTau pathology and requires biomarkers focused on brain vascular health. As attractive as the myriad biomarker possibilities are, their use needs to be tethered to a viable hypothesis for how a particular intervention may work. This can be complicated when testing diet patterns, in which some components may be related to an outcome more than others. Trials may have different study designs (selection of participants, outcomes, and interventions) if the goal is to influence cognitive aging vs dementia prevention. Even when dementia prevention is of interest, due to low incidence of this outcome until old age, cognitive and other secondary outcomes might be considered to capture aspects of brain aging.

4. Comparator group: In most trials the nutritional intervention is compared to participants' usual diet. Diet-as-usual, without any additional lifestyle counseling, is appealing as a straightforward option; however, this may not be an effective control, since participants would know nothing was changing. This poses additional limitations including potentially increased dropout rates for participants in the control group (due to dissatisfaction given the necessarily unblinded nature of the interventions)(2). Considering alternative diets that may be considered to have minimal effects in the study's outcomes may partially remedy these challenges (3, 4). Another approach could include network methods to develop new diet patterns which could serve as control diets that are different from the interventional patterns of interest. Information about the "nutritional environment" -the types and numbers of grocery stores or eateries in a region - may

also provide useful information about trial participants and their typical diet. Meal preparation may be required to assure adequate adherence.

5. Compliance: Biomarkers may eventually provide objective measures of participant compliance in nutrition studies. There may be some biomarkers that are sensitive to certain food consumption, and these are being measured recently through feeding studies. Researchers will also need to facilitate adherence, perhaps by providing components of the diet under study, like olive oil or berries. Dietary patterns that offer many ways to fulfill the diet could have a greater chance of being followed by participants. Network analysis can also help characterize compliance to a diet, or unintended consequences of some interventions; for example, if a network is "perturbed" by removing red meat in a diet intervention, this might inadvertently result in a decrease in leafy greens consumption if these foods are connected, or most often eaten with red meat. Network methods can also be expanded to track the amount of food consumed. However, the use of network methodologies in studying diet is new, and will need substantial research efforts to assess its value both broadly in epidemiologic research as well as in the context of randomized trials testing interventions.

6. Supplement Trials: Future trials of supplements could focus on effects in middle-aged people, in those with low baseline levels of the particular nutrient being investigated, the effects of giving suprathreshold doses, or cognitive or clinical staging, such as mild cognitive impairment, as an outcome. Standardization of reporting of adverse events, baseline characteristics, eligibility criteria, and methodology in general could also improve study design.

7. Novel Study designs:

a) Methodological Design of Relevance to Nutrition Studies: In considering nutrition-related trials, researchers need to ensure that the intervention can be defined, applied reliably across time, and across study sites. These considerations impact all aspects of study design. Challenges here include maintaining treatment fidelity over long periods of time as diet and pill-taking preferences can change over relatively brief periods. Randomized assignment to treatments and controls is essential to mitigate bias. Due to the complexity of many nutritional interventions, however, it may not be achievable to ensure double-blinding or allocation concealment, i.e., that blinded interventions are maintained. Enhancements or alternatives to the standard randomized, concurrent controlled trials include targeted designs, sequential designs (e.g., sequential multiple assignment randomization trials, or SMARTs); and cluster randomized trials, or stepped-wedge approaches. The latter two, although with limitations, may substantially enhance feasibility, recruitment, treatment efficiency, and reliability in that clusters or clinical sites are randomized rather than individuals at each site, allowing participants at each site to receive the same treatment.

As nutrition trials may generally involve complex interventions or multiple nutritional elements, they may also require multiple outcomes or endpoints in order to assess overall benefit. These may be outcomes from clinical, personal, social, and environmental perspectives. The assessment of endpoints or outcomes could require direct face-to-face interviews, remote or virtual interviews, remote self-administration, and minimally intrusive assessments. Determining a clear primary endpoint or outcome that encompasses objectively measurable clinical benefit is essential to avoid multiplicity and false discovery. Composite endpoints, here, might be considered.

There are different considerations for undertaking primary, secondary, and tertiary (i.e., symptomatic) prevention trials. True primary prevention trials are more formidable because they

require earlier and generally longer treatments, are based on the biology of the disease, and perhaps utilize less frequent assessments of outcomes than most trials typically require. Some degree of sample stratification would define various risk levels.

b) Sample sizes: The sample sizes of both prevention and symptomatic studies for nutritional interventions may vary greatly. Sample sizes, however, will be substantially influenced by the expected outcome, duration of a study, the ability of participants to comply with the intervention, and the type of outcome or endpoint used. For example, the longitudinal Three-City study in France measured baseline plasma levels of long-chain omega-3 fatty acids, and then assessed participants for dementia during a 17-year follow-up. This revealed an association between low levels of summed eicosapentaenoic acid (EPA) and docosahexaenoic acid (DHA) with higher dementia risk(5). The difference between the bottom and top long-chain omega-3 level quintiles was equivalent to adding 1.4 servings of fish to one's diet every week (equivalent to ≈750 mg/day EPA+DHA), and thus suggests a minimum dosage for a fish-eating intervention. The difference in dementia rates found also provided an effect size to estimate sample size for a future trial. As an example, based on these findings, more than 3,000 cognitively healthy older participants (aged 65+) would be needed in a 5-year trial comparing EPA+DHA versus placebo (with α=5% and 80% power) to show an effect size on dementia incidence equivalent to the 48% reduction in risk (95% CI 10%-58%) found between the top and bottom quintiles of summed EPA and DHA in the cohort. This effect size is likely not realistic, given what is currently known from interventions targeting dementia risk factors in general and diet in particular. Nevertheless, this example illustrates the much large sample size required to detect this effect compared to the smaller and short termed past neutral omega-3 cognition prevention clinical trials (6, 7). Thus, scalability becomes important, and broad eligibility criteria, e-health interventions, and e-health outcomes assessments may prove to be feasible for this. However, in elderly participants, e-health approaches may lead to a selection

bias; for example, selecting participants with high level of education, high cognitive reserve with lower probability for cognitive decline.

Other methodologic considerations include the decision to use and choice of biomarkers to support the primary outcome, and whether such nutritionally related biomarkers are in fact relevant to the intervention or to overall health status. Nutritional biomarkers may be used to characterize participants prior to treatment assignment; may serve as markers of treatment compliance or help to guide compliance or dosing in adaptive design trials; or more controversially as surrogate clinical outcomes markers. It is highly unlikely, however, that a change in a nutritional biomarker will have sufficient predictive value for clinical decline to be used as a surrogate outcome or endpoint in a larger phase III trial.

c) Trial designs: Approaches to nutrition trials would involve the consideration of several trial designs: targeted trials, platform studies, cluster randomized, stepped wedge, and futility trials. Targeted trials have a role when there is a nutritional characteristic or biomarker that is thought to predict the potential trial participants who would be responsive to the nutritional intervention compared to the control or placebo treatment. A straightforward example would be choosing participants with a vitamin deficiency for such a trial. More relevant to dementia might be the consideration of participants with a particular metabolic characteristic, for example *APOE* ε4 carriers with brain glucose hypometabolism for a dietary intervention expected to support brain energy metabolism (8). Targeted designs might be relatively more efficient than non-targeted designs with the right treatment and target, but their efficiency depends on the prevalence of the subgroup of people who do respond preferentially, and the distribution of the treatment effect across the subgroups.

Platform trials are trial designs in which multiple interventions can be compared simultaneously and/or sequentially under one protocol. These platforms may share control and placebo groups. They might use sequential interim analyses to help determine whether an intervention is ineffective and can be stopped, or due to uncertainty might be continued, potentially with a higher “dose” of a nutritional intervention. Importantly, new treatments are started on the platform, allowing contemporaneous overlap and sharing of control participants. Efficiency of recruitment may be gained because the several treatments are conducted under the same protocol. Along with interim analyses, however, come various amendments needed to adjust the interventions, sample size, dosing, allocations, or consent documents for the various treatments.

Examples of platform trials include:

i) Adaptive trial designs where post-randomization changes or adaptations need to have been planned and executed without undermining the validity and generalizability of the trial (9). Typical examples of adaptations include sample size reestimations that may be needed for inaccurate assumptions, group sequential designs, multi-arm multistage studies to allow treatment flexibility, interim futility, and efficacy analyses (10). Doing “run-ins” in which the intervention is slowly ramped up may help avoid enrolling people who are not committed or who don’t understand what the trial is about, however, this does reduce the external validity towards the wider target population. Adaptive trials may also present a more flexible trial design for nutrition and cognitive health trials: in these, changes are introduced gradually, and interventions can be tailored to an individual’s status.

ii) Cluster randomized trials compromise on the randomization of individual patients by identifying and randomizing existing groups (for example, residents of a specific assisted living facility, neighborhood, or clinical practice as in the PreDIVA trial (11)) into treatment or control. This approach can be substantially efficient. Risks of bias can be somewhat higher because some

participants and study staff may be aware of the design and the treatment they are receiving. This can be mitigated, however, by disguising the treatments, especially the controls, and using outcomes assessors who are blinded to the clinic group assignment, or even to the trial methods themselves.

iii) Stepped wedge, cluster randomized controlled trials employ cluster randomization but, rather than using parallel group assignment, the treatment clusters are made to crossover at fixed intervals or steps from control to intervention. The intervention is assigned across progressing time intervals so that delaying the start of the intervention can be used to help determine its effect. A prevention trial might then progress from an initial period in which few or none of the clusters receive the intervention, i.e., a delayed start to treatment, to more clusters crossing over to receive intervention after a certain lag time, with each step. In this way, clusters contribute outcomes under both control and intervention conditions.

8. Regional, cultural differences and underrepresented groups: Foods vary between different countries and cultures and more so in underrepresented groups. For example, cheese in France is not necessarily the same as cheese in the US, or fruit available in one country cannot be found in another. Even within a region, there is some variability between subjects according to the personalized interest in food and this can be a barrier leading to a decrease in intervention adherence. Because of this, researchers need measures that are sensitive to personal preferences, to the key components of these foods, such as fat content, and that can facilitate comparisons between studies conducted in different countries. More effort is also needed to not only enroll underrepresented groups in nutrition and cognitive health trials, but to understand their dietary habits. For future trials, researchers should explicitly identify the desired demographic characteristics of their study participants, recruitment goals should be clearly thought out prior to

study initiation, and they should be prepared to spend more than a year on recruitment. It is important to listen to the community to develop a deep understanding of its attitudes, as well as local family-related aspects, religiosity and spirituality. The physical presence of the investigators in the area may prove to be important facilitators and help increase trust.

Researchers should also use focus groups, with the help of trusted community partners, to streamline the recruitment protocols and understand what would help them meet recruitment goals. Initial engagements with a community should be focused on their concerns. For example, academic resources may be used to provide food or speakers of interest, even if completely unrelated to the study at hand. Once a study is underway, researchers should be prepared to provide the local community updates about the study's progress and findings. Mobile clinics or non-academic study centers may also be established to facilitate participation of people living far from academic centers, such as done in the current RURAL (Risk Underlying Rural Areas Longitudinal (RURAL)) Cohort study. In sum, researchers need to understand target demographics, carefully plan different aspects of the trial including much pilot work, consider family, religiosity and spirituality aspects of the population, utilize focus groups and work with trusted community leaders, use appropriate recruitment approaches and embed themselves via their personal presence within the community of interest to build their trust.

With the promise of electronic- and mobile-health approaches, it is important to identify a population that can use technology effectively. Though computer or mobile phone usage is not universal, it is growing: most people in the European Union have cell phones, as do an estimated 30-40% of people in sub-Saharan Africa, which is increasing rapidly. Yet care needs to be taken to not exclude underrepresented groups who may not have access to technology, or able to use it independently and who may be important target populations for interventions.

Recommendations for nutrition-based interventions: Following the group's discussions, two study design strategies emerged — one narrow and one broad. The narrow one targets interventions to a subset of people who are enriched for suboptimal diet and dementia risk, and/or who are at "nutritional risk" for cognitive decline with an expected large effect size. The broad design involves pragmatic interventions that are easy to deliver to a very large and diverse group of people but allows detection of smaller expected effect sizes. Technologies such as web-based "e-Health" or mobile phone-based "m-Health" apps may help scale up interventions, though a one-on-one coaching component may still be necessary. Both will face the difficulties of implementing a dietary intervention, which touches on an integral part of life that is complex and ingrained. Yet understanding whether or not people will follow the intervention in the real world is critical. In addition, timing of a nutrition trial in relation to disease development merits further study. Observational data can inform this question. Recent thinking lands on mid-life for optimal time window to modify the effects of a poor diet on the brain, as a time when risk factors for later dementia are active but reversible. This, however, requires intermediate surrogate outcomes predictive of future cognitive decline. Lessons from past trials should guide the design of new trials, though a one-size-fits-all standard for trial design is unlikely to work for future studies because they are likely to be very different from each other. For large trials, different randomization methods that can adapt to the nature of lifestyle interventions may be necessary. Studies also need to be sensitive to how dietary interventions play out in practice, and thus have adaptive yet principled ways of implementing these interventions or personalizing them.

References cited:

1. Pathak P, Helsley RN, Brown AL, Buffa JA, Choucair I, Nemet I, et al. Small molecule inhibition of gut microbial choline trimethylamine lyase activity alters host cholesterol and bile acid metabolism. American Journal of Physiology-Heart and Circulatory Physiology. 2020;318(6):H1474-H86.
2. Mirmiran P, Bahadoran Z, Gaeini Z. Common Limitations and Challenges of Dietary Clinical Trials for Translation into Clinical Practices. Int J Endocrinol Metab. 2021;19(3):e108170.
3. Martínez-Lapiscina EH, Clavero P, Toledo E, Estruch R, Salas-Salvadó J, San Julián B, et al. Mediterranean diet improves cognition: the PREDIMED-NAVARRA randomised trial. J Neurol Neurosurg Psychiatry. 2013;84(12):1318-25.
4. Liu X, Morris MC, Dhana K, Ventrelle J, Johnson K, Bishop L, et al. Mediterranean-DASH Intervention for Neurodegenerative Delay (MIND) study: Rationale, design and baseline characteristics of a randomized control trial of the MIND diet on cognitive decline. Contemp Clin Trials. 2021;102:106270.
5. Thomas A, Baillet M, Proust-Lima C, Féart C, Foubert-Samier A, Helmer C, et al. Blood polyunsaturated omega-3 fatty acids, brain atrophy, cognitive decline, and dementia risk. Alzheimers Dement. 2020.
6. Andrieu S, Guyonnet S, Coley N, Cantet C, Bonnefoy M, Bordes S, et al. Effect of long-term omega 3 polyunsaturated fatty acid supplementation with or without multidomain intervention on cognitive function in elderly adults with memory complaints (MAPT): a randomised, placebo-controlled trial. The Lancet Neurology. 2017;16(5):377-89.
7. Manson JE, Cook NR, Lee I-M, Christen W, Bassuk SS, Mora S, et al. Marine n−3 Fatty Acids and Prevention of Cardiovascular Disease and Cancer. New England Journal of Medicine. 2018;380(1):23-32.
8. Yassine HN, Finch CE. APOE Alleles and Diet in Brain Aging and Alzheimer’s Disease. Frontiers in aging neuroscience. 2020;12(150).
9. Bhatt DL, Mehta C. Adaptive Designs for Clinical Trials. New England Journal of Medicine. 2016;375(1):65-74.
10. Dimairo M, Pallmann P, Wason J, Todd S, Jaki T, Julious SA, et al. The Adaptive designs CONSORT Extension (ACE) statement: a checklist with explanation and elaboration guideline for reporting randomised trials that use an adaptive design. BMJ. 2020;369:m115.
11. Moll van Charante EP, Richard E, Eurelings LS, van Dalen JW, Ligthart SA, van Bussel EF, et al. Effectiveness of a 6-year multidomain vascular care intervention to prevent dementia (preDIVA): a cluster-randomised controlled trial. Lancet. 2016;388(10046):797-805.
